# The complete chloroplast genome of *Camellia huulungensis* Rosmann et Ninh, a golden *Camellia* species endemic to Vietnam

**DOI:** 10.1080/23802359.2024.2412227

**Published:** 2024-10-06

**Authors:** Xingwen Zhou, Fangyi Wang, Yiqing Xie, Jing Ning, Yuanfeng Xiao, Changjie Jiang, Guochang Ding, Yunxia Tang

**Affiliations:** a College of Architecture and Planning, Fujian University of Technology, Fuzhou, China; b Institute of Economic Forestry, Fujian Academy of Forestry, Fuzhou, China; c Golden Camellia Park of Nanning, Guangxi Zhuang Autonomous Region, Nanning, China; d College of Landscape Architecture and Art, Fujian Agriculture and Forestry University, Fuzhou, China

**Keywords:** *Theaceae*, *Camellia huulungensis*, chloroplast genome

## Abstract

*Camellia huulungensis* Rosmann & Ninh 1997, belonging to the sect. *Chrysantha*, holds important ornamental value and medicinal value. In this study, the complete chloroplast genome sequence of *C. huulungensis* was assembled using high-throughput sequencing technology. The entire length of chloroplast genome is 156,546 bp and contains a small single-copy region (18,257 bp), a large single-copy region (86,219 bp), and a pair of inverted repeat regions (26,035 bp). A total of 133 genes were annotated, including 88 protein-coding genes, 37 tRNA genes, and 8 rRNA genes. The overall GC content is 37.33%. The phylogenetic analysis showed that *C. huulungensis* is sister to *C. aurea*. The results can provide genetic data for further phylogenetic studies of *Camellia.*

## Instruction

The *Camellia* sect. Chrysantha Chang, known for the rare golden-yellow flowers, holds high ornamental value (Tang et al. 2023). Approximately, 52 species of golden camellias have been identified in southern China and Vietnam, with nearly 40 of these species naturally found in Vietnam (Manh et al. 2019). *Camellia huulungensis*, an endemic species in Vietnam, is a shrub that typically grows in valleys near tropical forests, thriving in the understory environment. The flowers and leaves of golden camellias are rich in beneficial natural compounds like tea polyphenols and flavonoids, making them prized for both ornamental and medicinal uses (He et al. 2018). Due to overharvesting for ornamental planting, the wild populations of golden camellias have been severely damaged (Hu et al. 2023). Consequently, all golden camellia species have recently been categorized as Critically Endangered, Endangered, or Near Threatened in the China Biodiversity Red List (data available at http://english.mep.gov.cn) (Wei et al. 2017). Additionally, frequent interspecific hybridization and polyploidy pose significant challenges for classification studies (Huang et al. 2014; Li et al. 2019). Therefore, developing molecular markers that can effectively distinguish among golden camellias is crucial for their breeding research.

The chloroplast genome is widely used in plant phylogenetic studies due to its highly conserved structure, moderate mutation rate, and lack of homologous recombination (Daniell et al. 2016). However, research on *C. huulungensis* has been limited due to its narrow distribution and small population size, and its chloroplast genome remains unknown. This study reports, for the first time, the complete chloroplast genome of *C. huulungensis*. The results provide valuable genomic resources that will aid in species identification, population genetics, and breeding research for golden *Camellia* species.

## Materials and methods

**Figure 1.**
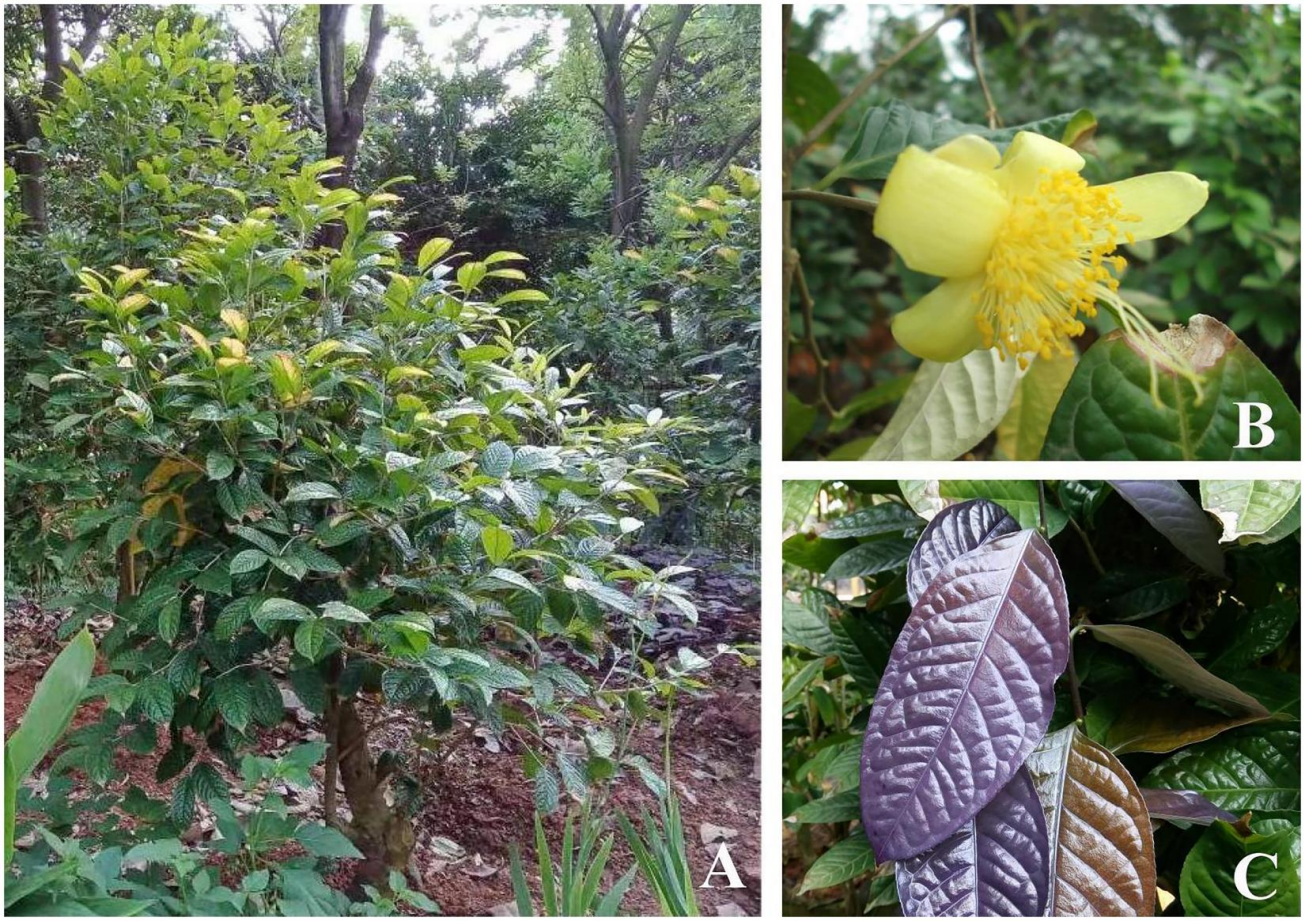
Natural ecological environment in the *Camellia huulungensis* Rosmann & Ninh planting area. The photograph was taken by Xingwen Zhou at Yulin Normal University in China (coordinates: 110.19E, 22.67 N) (A). The flowers are yellow, with 5–6 petals, and are either axillary or terminal (B). The leaves are narrow ovate or oblong, hairless, and have lightly serrated edges (C).

Species such as *C. huulungensis* were legally introduced from Southeast Asia and cultivated in the nursery of Yulin Normal University, China (N 22.67, E 110.19). These plants were originally obtained from Vietnam and have been cultivated for research purposes. Young leaves were collected and dried using silica gel (Figure 1), and the specimens were preserved in the Laboratory of Plant Specimen of Fujian University of Technology (Contact: Xingwen Zhou, E-mail: xingwenzhou2003@163.com), with the voucher specimen number ST202304012. Total DNA was extracted from fresh healthy young leaves of *C. huulungensis* by CTAB method (Li et al. 2013). The extracted total DNA was sent to BGI Technology Service Co., Ltd. (Wuhan, China) to construct library, and the complete chloroplast genome was sequenced using the Illumina Hiseq 4000 sequencing platform with a paired-end read length of 150 bp. We used Trimmomatic to filter the raw sequencing reads, removing low-quality reads and adapter sequences. Bowtie2 was employed to align the clean reads and extract chloroplast plastome reads. The reads were then assembled into the complete chloroplast plastome using GetOrganelle v.3.11.0 (Jin et al. 2020), with the parameters set to –w 95 –R 20 –k 21, 35, 45, 55, 65, 75 –F embplant_pt. The PGA (Qu et al. 2019) was used for annotation, with *Camellia flava* (OR605723) serving as the reference sequence. Annotation results were confirmed using GB2sequin (Tillich et al. 2017). Visualization of the chloroplast genome, cis-splicing and trans-splicing genes was done using CPGView (Liu et al. 2023). The assembled chloroplast genome and its detailed annotations were submitted to GeneBank with the accession number PP531447.

To explore the phylogenetic position of *C. huulungensis*, we downloaded the chloroplast genome sequences of 39 *Camellia* species from the National Center for Biotechnology Information (NCBI) database (https://www.ncbi.nlm.nih.gov/). Two *Polyspora* species were used as an outgroup. The whole chloroplast genome sequences were aligned used MAFFT v7.505 (Katoh and Standley 2016). We used the online tree-building platform CIPRES Science Gateway V 3.3 (Miller et al. 2010) to construct phylogenetic trees using three methods: Maximum Likelihood (ML) with RAxML-HPC2 on XSEDE 8.2.12, Maximum Parsimony (MP) with PAUP on XSEDE 4.a165, and Bayesian Inference (BI) with MrBayes on XSEDE 3.2.7a. For the ML tree construction, we used the TVM+F + I + I + R5 model for nucleotide substitution, employed the Bootstrap algorithm with 1000 replicates, and kept the remaining parameters at their default values

## Results

**Figure 2.**
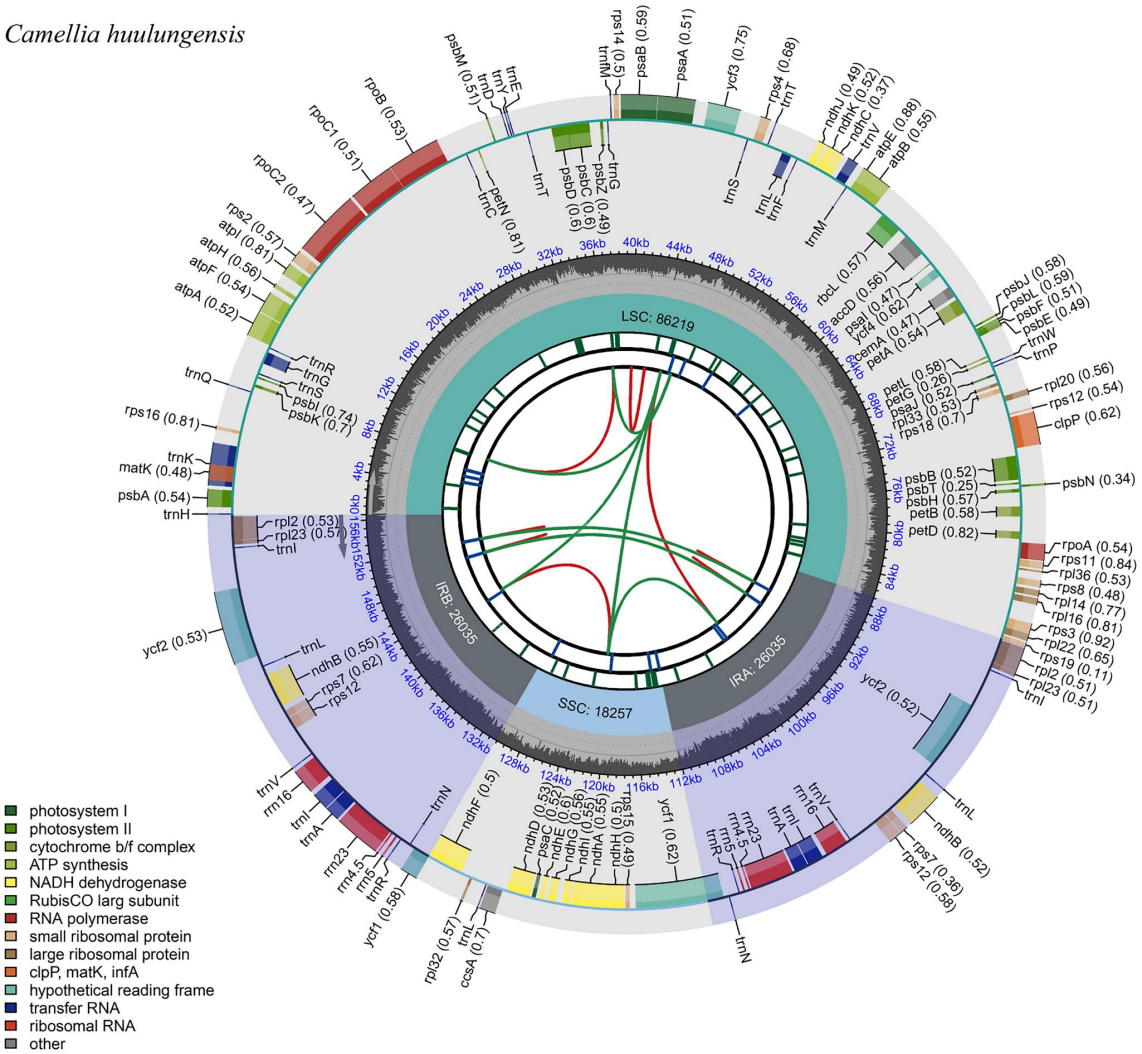
Gene map of the complete chloroplast genome of *Camellia huulungensis*. The species name is shown in the top left corner. The map contains six tracks. From the center outward, the first track shows the dispersed repeats. The dispersed repeats consist of direct (D) and palindromic (P) repeats, connected with red and green arcs. The second track shows the long tandem repeats as short blue bars. The third track shows the short tandem repeats or microsatellite sequences as short bars with different colors. The colors, the type of repeat they represent, and the description of the repeat types are as follows. Black: c (complex repeat); green: p1 (repeat unit size = 1); yellow: p2 (repeat unit size = 2); purple: p3 (repeat unit size = 3); blue: p4 (repeat unit size = 4); orange: p5 (repeat unit size = 5); Red: p6 (repeat unit size = 6). The small single-copy (SSC), inverted repeat (IRa and IRb), and large single-copy (LSC) regions are shown on the fourth track. The GC content along the genome is plotted on the fifth track. The genes are shown on the sixth track. Genes are color-coded by their functional classification. The transcription directions for the inner and outer genes are clockwise and anticlockwise, respectively. The functional classification of the genes is shown in the bottom left corner.

The chloroplast genome of *C. huulungensis* was successful assembled with an average depth of 671.57× (Figure S1). The complete chloroplast genome of *C. huulungensis* is a typical circular double-stranded DNA molecule with a length of 156,546 bp (Figure 2). The cp genome had a typical quadripartite structure of most angiosperms, consisting of a large single-copy (LSC, 86,219 bp), a small single-copy (SSC, 18,257 bp), and two inverted repeats (IRs, 26,035 bp). The GC content of *C. huulungensis* was different in different regions, the total GC content was 37.33%, and the GC content of IR region, LSC and SSC was 42.97, 35.35 and 30.6%, respectively. A total of 133 genes were identified in the cp genome of *C. huulungensis*, including 88 protein-coding genes (PCGs), 37 transfer RNA genes (tRNA) and eight ribosomal RNA genes (rRNA). Nine protein-coding genes (*atpF, ndhA, ndhB, petB petD, rpl2, rpl12, rpoC1, rps16*) and six tRNA (*trnA-UGC, trnG-GAU, trnG-UCC, trnK-UUU, trnL-UAA, trnV-UAC*) contained an intron (Figure S2). Two genes (*clpP and ycf3*) each have two introns. In addition, *rps12* was identified as a trans-splicing gene (Figure S3).

**Figure 3.**
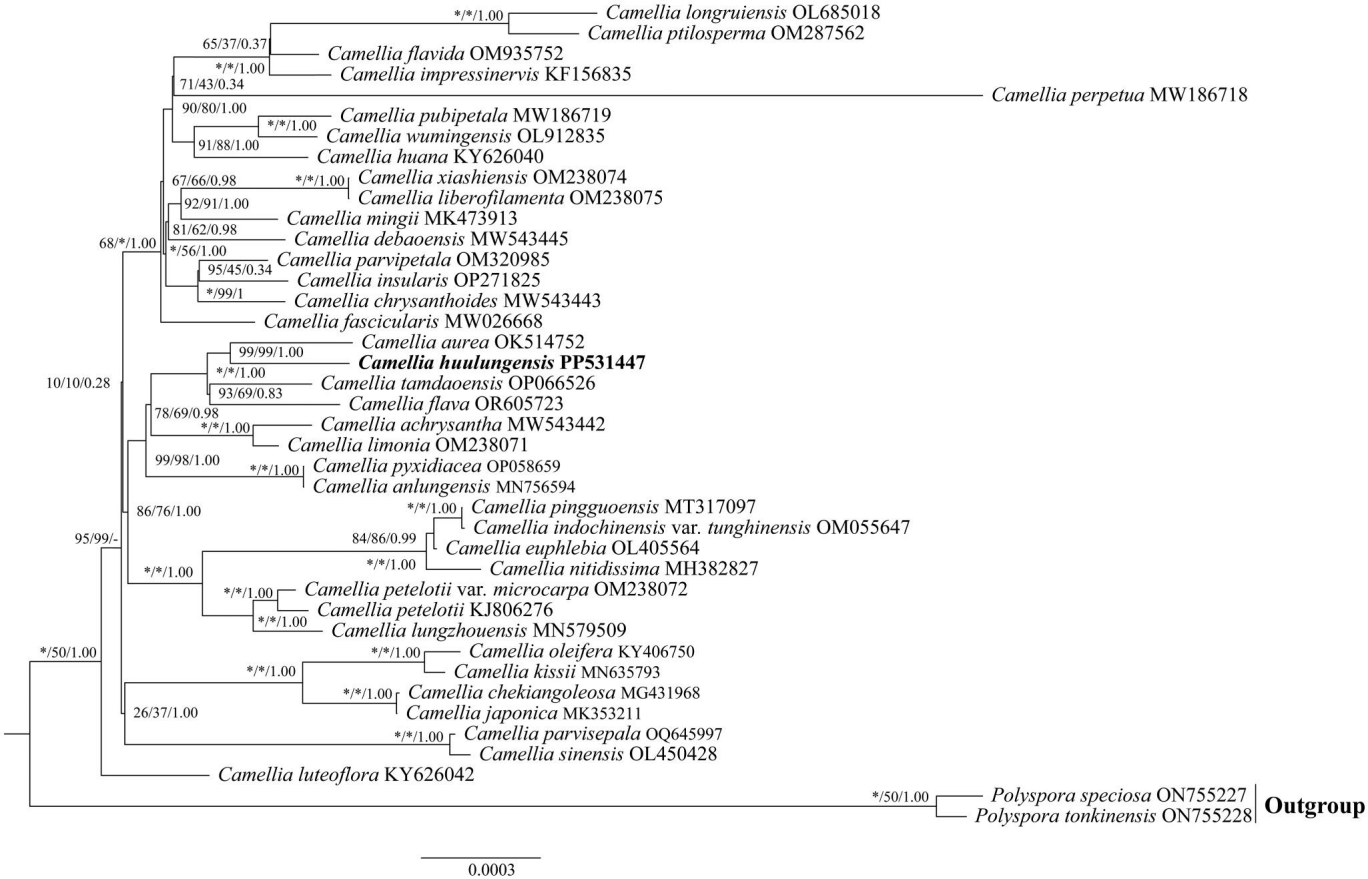
The phylogenetic tree of 38 *Camellia* species and 2 outgroup species was obtained by maximum likelihood (ML) analysis based on complete plastome sequences. The numbers near the nodes represent bootstrap percentages and Bayesian posterior probabilities (BP_ML_, BP_MP_, PP); *Indicates nodes with a 100 bootstrap, while – indicates inconsistencies among ML, MP, and BI trees. The scale represents the number of nucleotide substitutions at each site. The sequences used for tree construction are as follows: *Camellia achrysantha* (MW543442) (Lai and Tang 2021), *C. anlungensis* (MN756594) (Zhu et al. 2020), *C. aurea* (OK514752), *C. chekiangoleosa* (MG431968) (Yin et al. 2021), *C. chrysanthoides* (MW543443) (Lai and Tang 2021), *C. debaoensis* (MW543445) (Zheng and Wei 2021), *C. euphlebia* (OL405564), *C. fascicularis* (MW026668) (Ding et al. 2022), *C. flava* (OR605723), *C. flavida* (OM935752), *C. huana* (KY626040) (Wang et al. 2017), *C. huulungensis* (PP531447), *C. impressinervis* (KF156835),), *C. indochinensis* var. *tunghinensis* (OM055647), *C. insularis* (OP271825), *C. japonica* (MK353211) (Li et al. 2019), *C. kissii (MN635793)* (Cao et al. 2020), *C. liberofilamenta* (OM238075) (Wang et al. 2017), *C. limonia* (OM238071) (Ding et al. 2022), *C. lungzhouensis* (MN579509) (Fan et al. 2021), *C. luteoflora* (KY626042) (Wang et al. 2017), *C. mingii* (MK473913) (Zhang et al. 2019), *C. nitidissima* (MH382827), *C. oleifera* (KY406750) (Liang et al. 2024), *C. parvipetala* (OM320985), *C. parvisepala* (OQ645997), *C. perpetua* (MW186718) (Pei et al. 2020), *C. petelotii* (KJ806276) (Huang et al. 2014), *C. petelotii* var. *microcarpa* (OM238072), *C. pingguoensis* (MT317097), *C. ptilosperma* (OM287562), *C. pubipetala* (MW186719) (Fan et al. 2021), *C. pyxidiacea* (OP058659) (Ran et al. 2024), *C. longruiensis* OL685018, *C. sinensis* (OL450428) (Chen et al. 2023), *C. tamdaoensis* (OP066526), *C. wumingensis* (OL912835), *C. xiashiensis* (OM238074) (Ding et al. 2022). *Polyspora speciosa* (ON755227), *Po. tonkinensis* (ON755228).

A total of 40 complete plastome sequences, were utilized to conduct phylogenetic analyses and elucidate the phylogenetic positions of *C. huulungensis* (Figure 3). The phylogenetic trees generated using the ML, MP, and BI methods produced a consistent topology, with high bootstrap values (bootstrap ≥99 and posterior probability = 1). According to the phylogenetic trees, *Camellia* is a monophyletic group, and *C. huulungensis* is sister to *C. aurea*.

## Discussion and conclusion

In this study, the complete chloroplast genome of *C. huulungensis* was successfully sequenced and assembled. The results demonstrate that the chloroplast genome exhibits a highly conserved structure. The genomic structure of *C. huulungensis* consists of SSC, LSC, and a pair of IRs. The structure and gene content of the chloroplast genome are similar to those of other published chloroplast genomes in the sect. *Chrysantha* (Ding et al. 2022). Phylogenetic analysis revealed the phylogenetic position of *C. huulungensis* within the *Camellia* sect. *Chrysantha*. Additionally, it showed that *C. huulungensis* has a close genetic relationship with *C. aurea*. These findings not only augment the chloroplast genome data available for the *Camellia* sect. *Chrysantha* but also provide a theoretical foundation for the future conservation and rational utilization of *C. huulungensis* and other species within this section. Further research can delve into the differences and commonalities between *C. huulungensis* and *C. aurea* in terms of morphological characteristics, physiological traits, and ecological adaptability, thereby uncovering more secrets about the evolution and diversity formation of *Camellia* species.

All species within the *Camellia* sect. *Chrysantha* are classified as second-class national key protected wild plants in China, possessing high ornamental value and significant potential for pharmaceutical development. The chloroplast genome harbors a wealth of genetic information that can be leveraged for various applications; however, there is currently limited research on the chloroplast genomes of plants in this section both domestically and internationally. Thus, elucidating the chloroplast genome of *C. huulungensis* to investigate its genetic evolution and phylogeny is of paramount importance. In conclusion, this study presents the first complete chloroplast genome of *C. huulungensis*, providing a valuable reference for future research on chloroplast genomes. This data can be leveraged in comparative studies and the development of molecular markers for precise species identification.

## Supplementary Material

FigureS1.jpeg

FigureS3.jpg

FigureS2.jpg

## Data Availability

The genome sequence data that support the findings of this study are openly available in GenBank of NCBl at https://www.ncbi.nlm.nih.gov/ under accession No. PP531447. The associated BioProject, SRA, and Bio-Sample numbers are PRJNA1089488, SRR28385169 and SAMN40540877, respectively.
