# Supplementary figures and images for: The complete chloroplast genome of Camellia huulungensis Rosmann et Ninh, a golden Camellia species endemic to Vietnam
Source: Mitochondrial DNA B Resour. 2024 Oct 6;9(10):1365–9. doi: 10.1080/23802359.2024.2412227 (PMC11459732; doi:10.1080/23802359.2024.2412227)

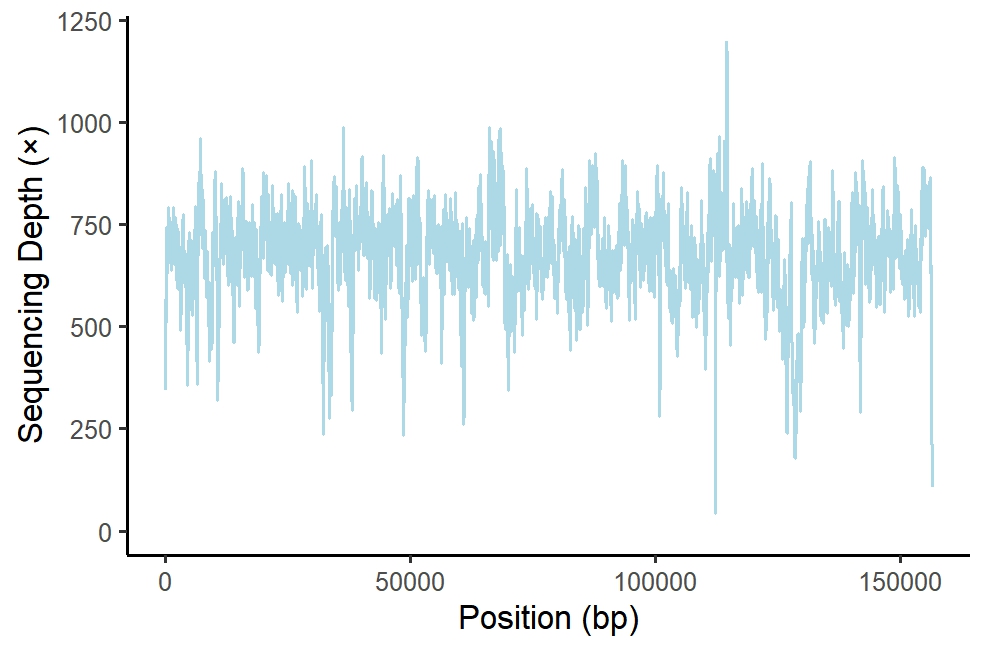

Supplement: FigureS1.jpeg [file TMDN_A_2412227_SM1812.jpeg]

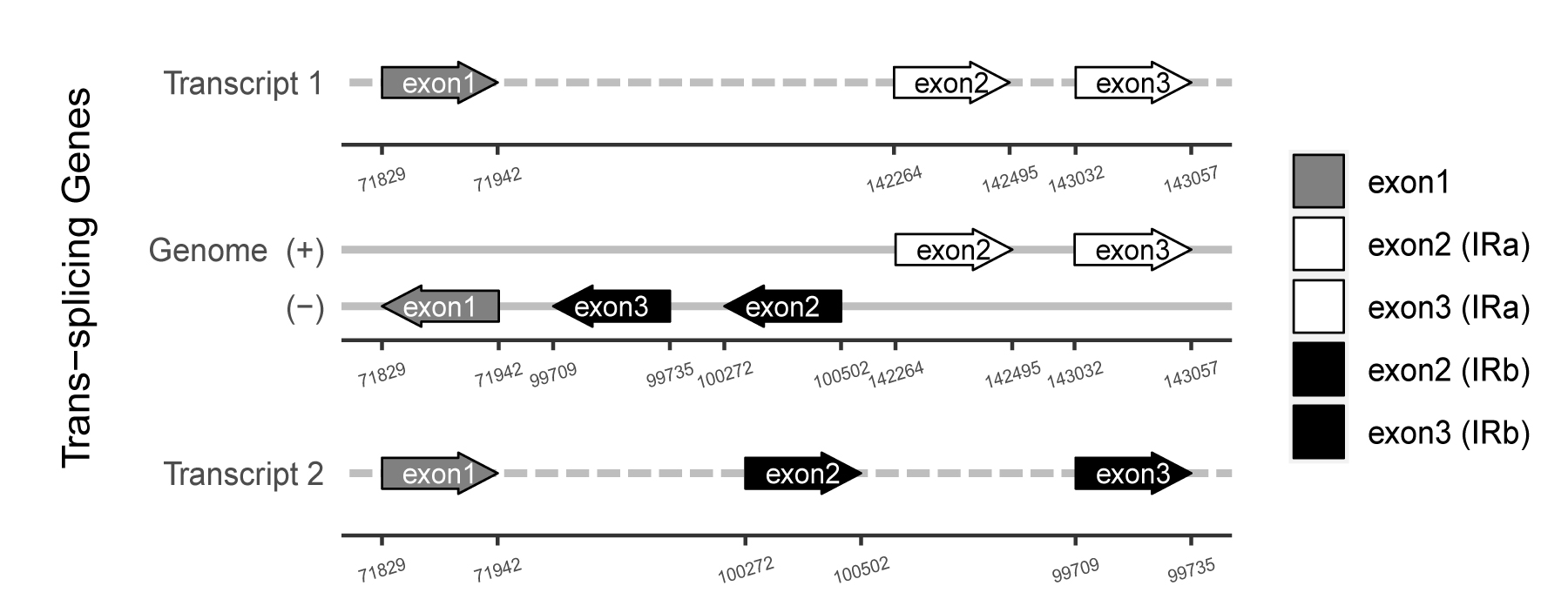

Supplement: FigureS3.jpg [file TMDN_A_2412227_SM1811.jpg]

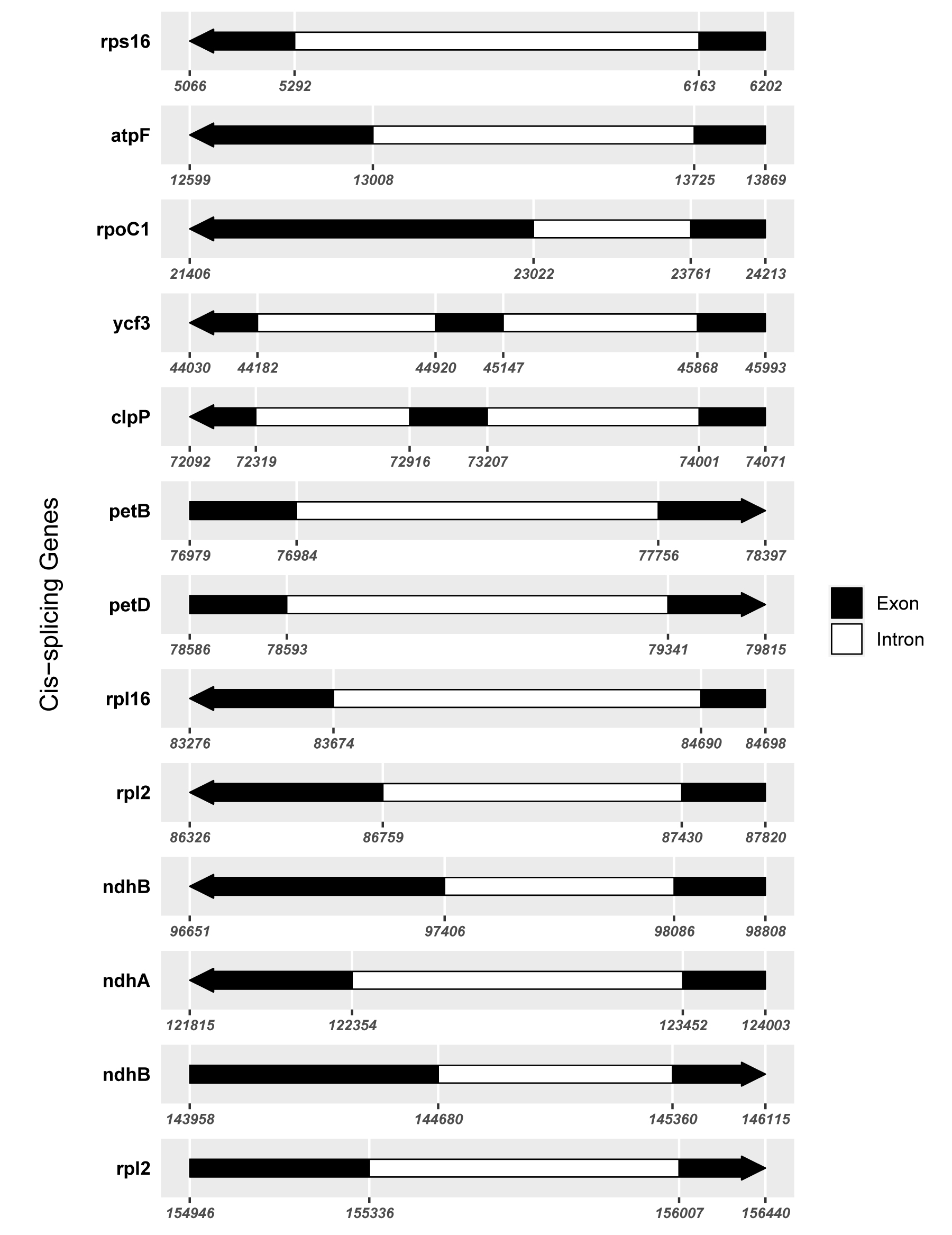

Supplement: FigureS2.jpg [file TMDN_A_2412227_SM1810.jpg]
